# Supplementary material for: Chromosomal instability in aneuploid acute lymphoblastic leukemia associates with disease progression
Source: EMBO Mol Med. 2023 Dec 15;16(1):64–92. doi: 10.1038/s44321-023-00006-w (PMC10897411; doi:10.1038/s44321-023-00006-w)
Supplement: Supplementary file 1 — Appendix [file 44321_2023_6_MOESM1_ESM.pdf]

APPENDIX FIGURES AND TABLES

Appendix Figure S1

Appendix Table S1

Appendix Table S2

Appendix Table S3

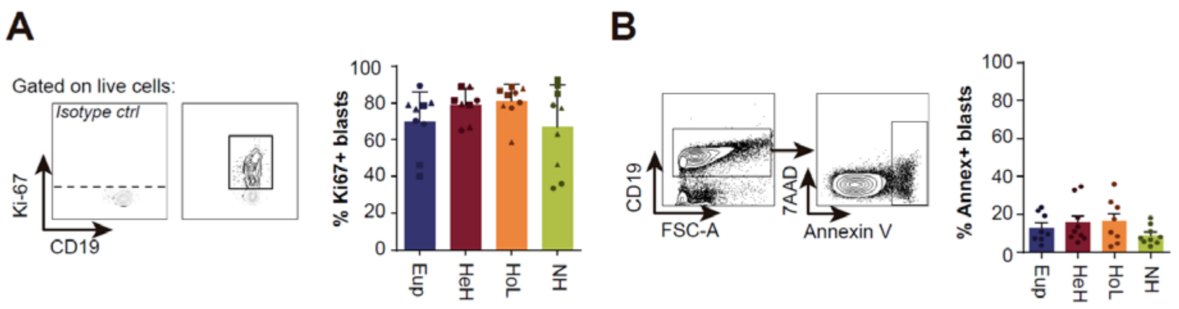

Appendix Figure S1 (Related to Figure 2). (A) *Left*, representative FACS staining of Ki-67 and CD19 in PDX samples. *Right*, percentage of Ki-67+ blasts (CD19+) in the indicated ploidy groups (n=8 Eup-, 9 HeH-, 9 HoL- and 9 NH-B-ALL). (B) *Left*, representative FACS staining of annexin V, 7AAD and CD19 in PDX samples. *Right*, percentage of annexin V and 7AAD positive+ blasts (CD19+) in the indicated ploidy groups (n as above). Mean value of each PDX is shown and error bars represent the SEM. Symbols (circles, triangles, squares) represent individual PDX samples analysed in each group; two-way ANOVA or Student's t test.

ID	Technique	Time point	Karyotype
	G-banding	Primary	56-59, XX + X,+4,+5,+6,+7,+8,+10,+14,+14,+14,+15,+17,+18, +21,+21,+22 [cp6]
HeH#1	scWGS	Primary	58-60, XX, +X, +4, +6, +7, +8, +10, +14, +14, +17, +18, +21, +21, +22 [cp14]
	M-FISH	PDX	53-59, XX, +X, +4, +6, +7, +8, +10, +14, +14, +17, +18, +21, +21, +22 [cp20]
	G-banding	Primary	57-58 XX, +X +X, +3 ,+ 6, +8, +8, +10, +10, +13, +14,+14, +17, +18,+18, +21, +21 [cp13]
HeH#2	scWGS	Primary	48-61, XX, +X, +X, +3, +6, +8, +10, +14, +17, +18, +18, +21, +21 [cp12]
	M-FISH	PDX	56-58, XX, +X, +X, +3, +6, +8, +10, +14, +17, +18, +18, +21, +21 [cp20]
	G-banding	Primary	54,XX,+X,+6,+8,+14,+17,+18,+21,+21[15] / 54,idem,-13,+mar[11] / 46,XX[19]
HeH#3	scWGS	Primary	na
	M-FISH	PDX	52-57, XX, +X, +4, +6, +8, +14, +17, +18, +21, +21 [cp20]
	G-banding	Primary	46,XX[18]/32-38,XX,-3,-5,-6,-7,-10,-13,-18,-20[6]/50-54,XX,+17,+18,inc[4]
HoL#1	scWGS	Primary	na
	M-FISH	PDX	52-55, XX, +X, +6, +8, +10, +14, +17, +18,+21, +21 [cp20]
	FISH/DI	Primary	nuc ish (MLLx1-3, BCR-ABLx1-3, ETV6/RUNX1x1-4)[200] / DI=0.76
HoL#2	scWGS	Primary	35-41, X, -X, -2, -3, -7, -9, -12, -13, -14, -16, -17 [cp11]
	M-FISH	PDX	49-61, XX, +1, +4, +6, +8, +8, +8, +8, +10, +11, +15, +19, +20, +20, +21, +21 [cp20]
	G-banding	Primary	37,XX,-2,-3,-4,-7,-12,-13,-15,-16,-17[6]/73,XXX,+X,+1,-2,-3,-4,+5,+6,-7,+8,+9,+11,-12,-13,+14,-15,-16,-17,+18,+19,+20,+21,+22[8]
HoL#3	scWGS	Primary	37-38, XX, -2, -3, -4, -7, -12, -13, -15, -16, -17 [cp10]
	M-FISH	PDX	59-64, XX, +X, +X, +1, +1, +5, +6, +6, +10, +11, +18, +19, +20, +21, +22 [cp20]
	G-banding	Primary	27<1n>,XX,+14,+18,+21[20]
NH#1	scWGS	Primary	54, XX, +X, +X, +14, +14, +18, +18, +21, +21
	M-FISH	PDX	27<1n>, XX, +14, +18, +21 [16]/54, XX, +X, +X, +14, +14, +18, +18, +21, +21[17]
	G-banding	Primary	52,XY,+X,+Y,+14,+14,+21,+21[8]/46,XY[2]
NH#2	scWGS	Primary	na
	M-FISH	PDX	52, XY, +X, +Y, +14, +14, +21, +21
	FISH	Primary	nuc ish (2,3,5,7,8,9,11,12,13,14,15,16,17,22)x1 (<30 chr)
NH#3	scWGS	Primary	57-59, XY, +X, +X, +6, +6, +10, +10, +18, +18, +21, +21 [cp7]
	M-FISH	PDX	47-56, XY, +X, +X, +6, +6, +10, +10, +18, +18, +21, +21 [cp14]

Appendix Table S1: Karyotypes of aneuploid cB-ALL samples as assessed by different techniques before (primary) and after PDX expansion. DI: DNA index; M-FISH: Multicolor Fluorescence *in situ* Hybridization; na: not-assessed; PDX: patient-derived xenograft; scWGS: single-cell Whole Genome Sequencing;

Chromosome ID	Fitness cost B-ALL (α)	density mutated genes - COSMIC database (β)	TSG/EG/OG added density (γ)	Features affecting cell functioning	Centromere size (Mb)	Length (Mb)	Struc. Features affecting chr. Segregation	Chromosome contribution to cell fitness (Phi)
1	0.079497908	0	0.041838843	0.040445584	12.42	247.249719	0.074879227	0.115324811
2	0.054393305	0	0.052364865	0.035586057	9.34	242.951149	0.065310493	0.100896549
3	0.050209205	0	0.038572806	0.029594004	9.17	199.501827	0.06434024	0.093934244
4	0.041841004	0	0.031029619	0.024290208	5.09	191.273063	0.088408644	0.112698852
5	0.050209205	0	0.042959427	0.031056211	4.77	180.857866	0.113207547	0.144263758
6	0.058577406	0.134615385	0.044132397	0.079108396	6.05	170.899992	0.110743802	0.189852198
7	0.058577406	0.221153846	0.038506418	0.106079223	5.89	158.821424	0.118845501	0.224924724
8	0.054393305	0.057692308	0.036507937	0.049531183	4.52	146.274826	0.119469027	0.16900021
9	0.062761506	0	0.030913978	0.031225162	5.06	140.273252	0.130434783	0.161659944
10	0.037656904	0	0.037089872	0.024915592	4.03	135.374737	0.158808933	0.183724525
11	0.062761506	0.221153846	0.033653846	0.1058564	5.29	134.452384	0.200378072	0.306234471
12	0.029288703	0.134615385	0.060422961	0.074775683	3.54	132.349534	0.234463277	0.30923896
13	0.025104603	0.048076923	0.059405941	0.044195822	1.6	114.14298	0.21875	0.262945822
14	0.046025105	0.019230769	0.060344828	0.0418669	1.56	106.368585	0.442307692	0.484174593
15	0.016736402	0	0.051660517	0.022798973	1.7	100.338915	0.447058824	0.469857796
16	0.033472803	0.086538462	0.046632124	0.055547796	3.82	88.827254	0.282722513	0.33827031
17	0.071129707	0.038461538	0.055353902	0.054981716	2.22	78.774742	0.720720721	0.775702437
18	0.020920502	0.038461538	0.030769231	0.030050424	1.61	76.117153	0.260869565	0.290919989
19	0.087866109	0	0.036621824	0.041495977	2.85	63.811651	0.807017544	0.848513521
20	0.012552301	0	0.049713193	0.020755165	2.71	62.435964	0.372693727	0.393448892
21	0	0	0.046948357	0.015649452	1.23	46.944323	0.471544715	0.487194168
22	0.020920502	0	0.028985507	0.016635336	1.18	49.691432	0.940677966	0.957313303
X	0.025104603	0	0	0.008368201	5.94	154.913754	0.101010101	0.109378302

Appendix Table S2: Values used to calculate the fitness cost of specific aneusomies in Figure 6B.

Parameter	Description	Value
Δt	Time step	2h
N_0	Initial cell number	500 cells
K	Carrying capacity	10^6 cells
P_{DIV}	Probability of cell division	[0.214, 0.4] day^{-1}
P_{CIN}	Probability of missegregation	P_{DIV} [0, 0.4] day^{-1}
P_{WGD}	Probability of endoreplication	0.11 P_{DIV} day^{-1}
P_{DEATH}	Probability of death	P_{DIV}/10 day^{-1}
σ	Selection strength coefficient	[0, 1]

Appendix Table S3. Parameters used for agent-based modelling.
