# Supplementary material for: Chromosomal instability in aneuploid acute lymphoblastic leukemia associates with disease progression
Source: EMBO Mol Med. 2023 Dec 15;16(1):64–92. doi: 10.1038/s44321-023-00006-w (PMC10897411; doi:10.1038/s44321-023-00006-w)
Supplement: Supplementary file 10 — Expanded View Figures [file 44321_2023_6_MOESM10_ESM.pdf]

Expanded View Figures

Figure EV1. Aneuploid cB-ALL show higher rates of mitotic defects and chromosomal clonal heterogeneity in PDX models.

(**A**) Box-plots representing the percentage of mitotic defects in the indicated B-ALL PDX samples ($n = 36$ PDX, $n = 3$ PDX per primary sample; $n = 200$ mitotic cells per PDX sample, total = 7200 mitotic cells). The box begins in the first quartile (percentile 25%) and ends in the third quartile (percentile 75%), central horizontal line represents the median value. Vertical line represents segment of furthest data from minimum (bottom) to maximum (top) values. (**B**) Frequency of mitotic PDX-expanded primary blasts from (**A**) with the indicated mitotic defects (n = 36, 3 cB-ALL-PDX samples per leukemia; $n = 200$ mitotic cells per PDX sample, Total=7200 mitotic cells). (**C**) Box-plots representing the percentage of late mitosis defects in the indicated cB-ALL PDX samples ($n = 36$ PDX samples; $n = 123$ Eup, 116 HeH, 163 HoL and 186 NH late mitoses). The box begins in the first quartile (percentile 25%) and ends in the third quartile (percentile 75%), central horizontal line represents the median value. Vertical line represents segment of furthest data from minimum (bottom) to maximum (top) values. (**D**) Chromosome number distributions of cells in the indicated B-ALL PDX samples as determined by M-FISH on metaphase spreads. Center values indicate the median and error bars indicate the SEM. Number of cells analyzed and chromosome modal numbers (MN) are indicated at the top. Dashed line shows the normal euploid chromosome number. (**E**) Chromosome number distributions of cells in matching primary and PDX samples, as determined by scWGS (blue) and M-FISH (red). Center values indicate the median and error bars indicate the SEM. (**F**) Chromosome MN in the indicated B-ALL PDX samples as determined by M-FISH analyses from (**D**). Red dashed lines show the disomic (2n) copy-number. (**G**) Box-plots representing the variability of chromosome copy-numbers observed by M-FISH analyses from (**D**), as determined by the standard deviation (SD) in the indicated ploidy group. The box begins in the first quartile (percentile 25%) and ends in the third quartile (percentile 75%), central horizontal line represents the median value. Vertical line represents segment of furthest data from minimum (bottom) to maximum (top) values.

▶

© The Author(s)

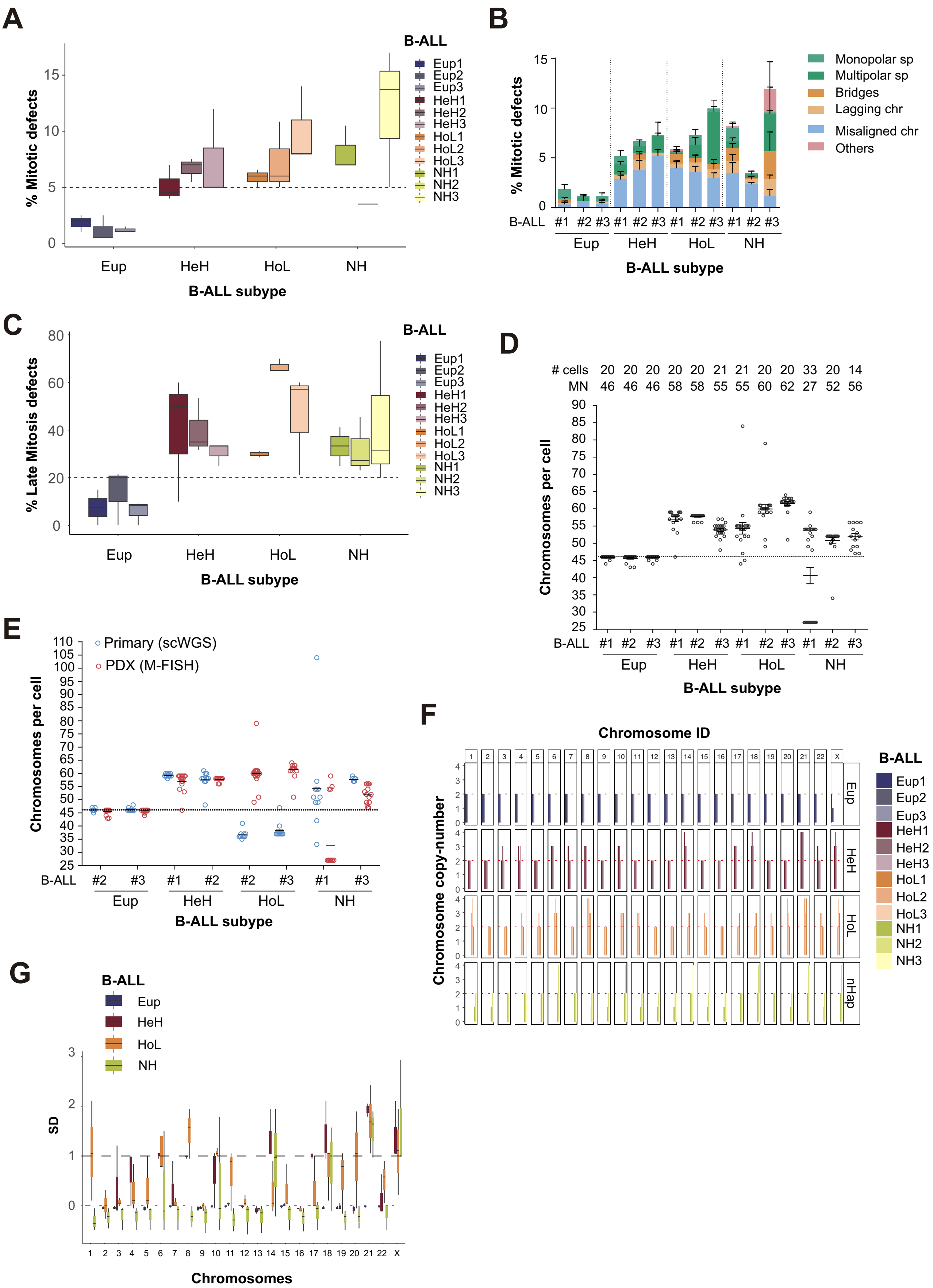
A
B-ALL
% Mitotic defects
B-ALL subype
Eup HeH HoL NH
Eup1 Eup2 Eup3 HeH1 HeH2 HeH3 HoL1 HoL2 HoL3 NH1 NH2 NH3
B
Monopolar sp
Multipolar sp
Bridges
Lagging chr
Misaligned chr
Others
B-ALL subype
C
% Late Mitosis defects
D
cells 20 20 20 20 20 21 21 20 20 33 20 14
MN 46 46 46 58 58 55 55 60 62 27 52 56
Chromosomes per cell
E
Primary (scWGS)
PDX (M-FISH)
F
Chromosome ID
Chromosome copy-number
nHap
G
SD
Chromosomes

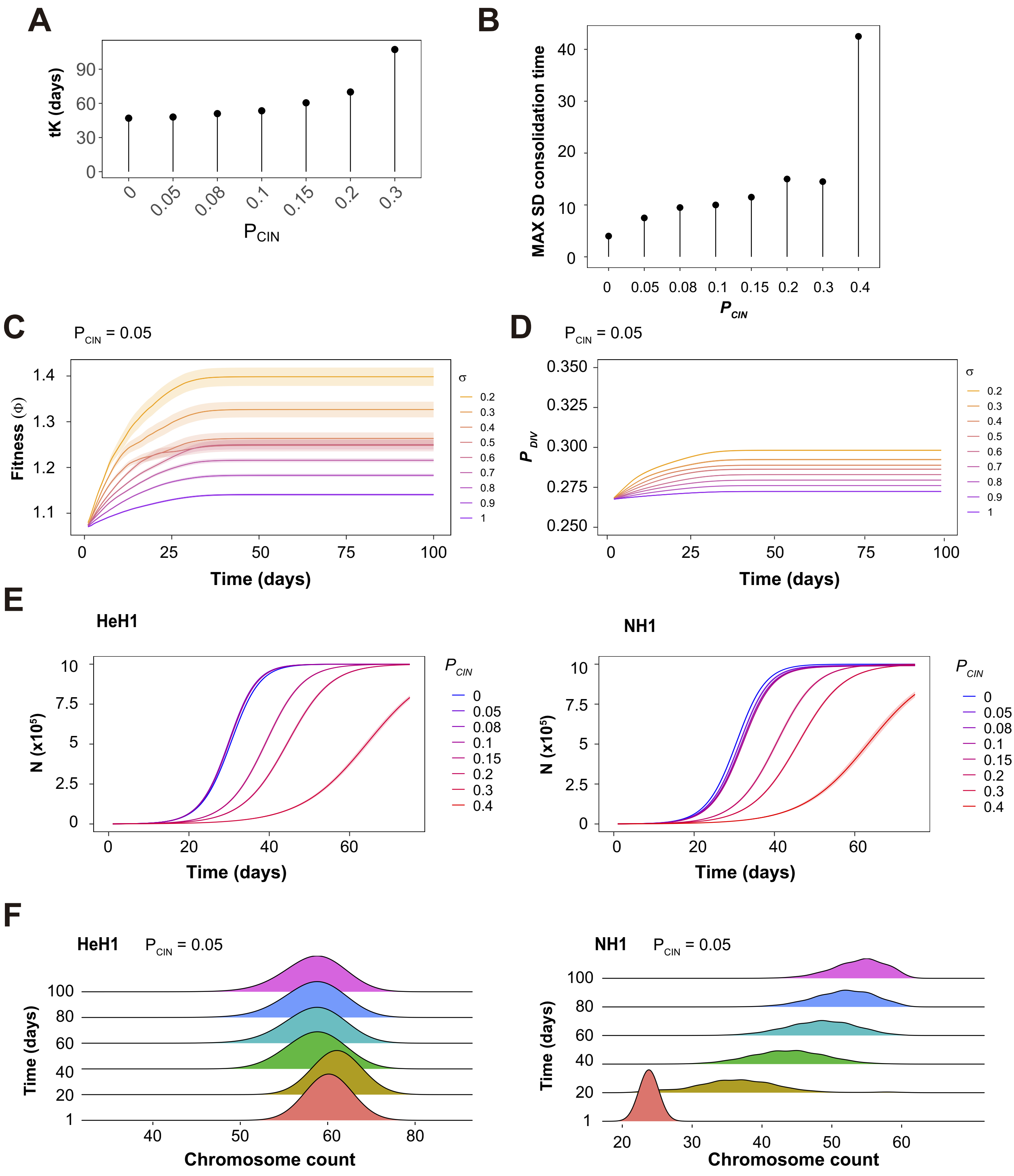

Figure EV2. Mathematical modeling associates low-to-mid levels of CIN as drivers of clonal heterogeneity and disease progression in cB-ALL.

(**A**) Average time to reach carrying capacity (tK) at the indicated CIN levels ($N = 50$ simulations). (**B**) Time to consolidate a stable karyotype at the indicated CIN levels ($N = 50$ simulations). (**C**) Simulated dynamics of average fitness at the indicated CIN levels ($N = 50$ simulations). (**D**) Simulated dynamics of cell division rates at the indicated CIN levels ($N = 50$ simulations). (**E**) Simulated cell numbers for virtual sample HeH1 (left) and NH1 (right) at the indicated CIN levels ($N = 20$ simulations). Average fitness is plotted relative to that expected for a euploid cell, the latter being equal to 1. (**F**) Karyotype variability as observed by chromosome counts at the indicated time points from in silico simulations with the virtual HeH1 (left) and NH1 (right) samples at moderate CIN levels ($P_{CIN} = 0.05$).

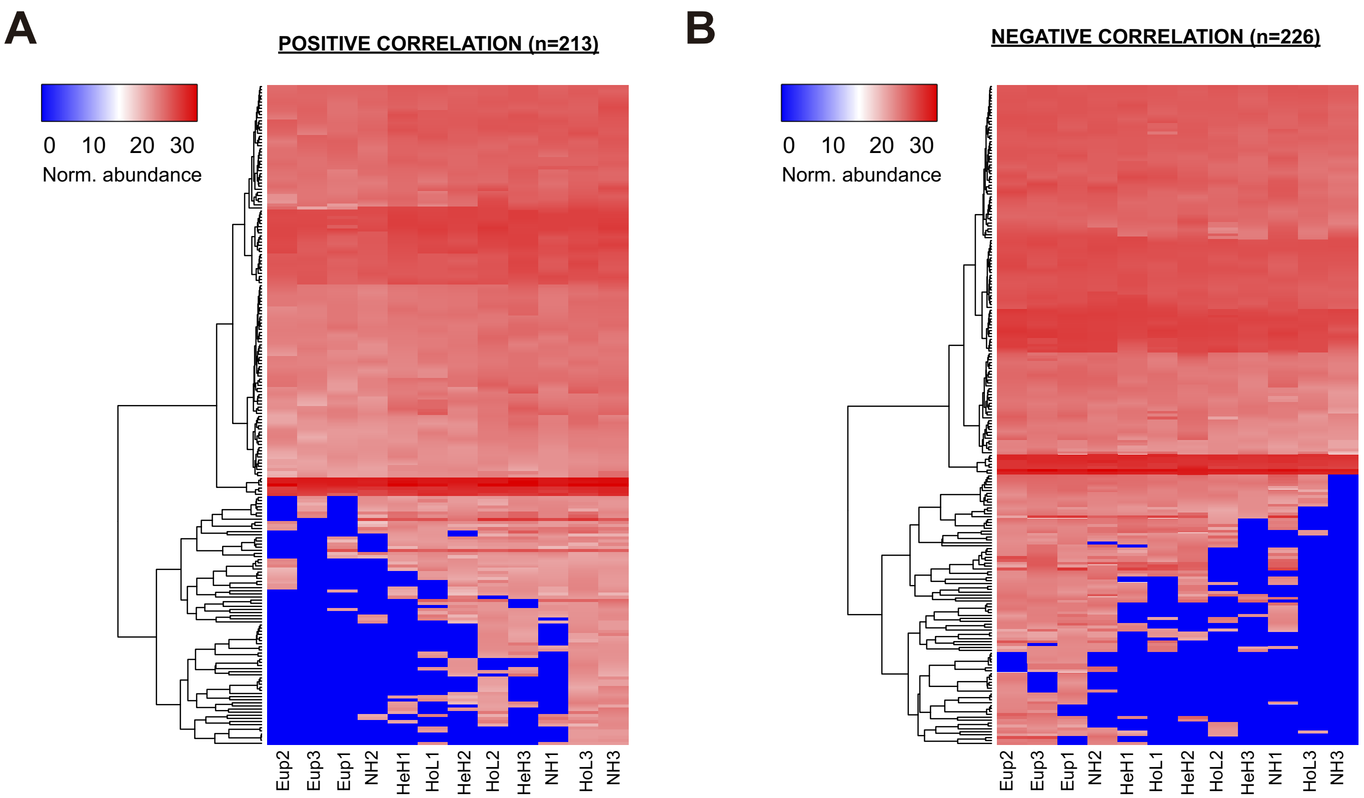

Figure EV3. Proteins associated with CIN in cB-ALL.

(**A**, **B**) Heatmaps depicting proteins positively (**A**) and negatively (**B**) correlated with CIN. Protein abundances in whole-cell lysates are color-represented in the legends (top). Pearson correlation coefficient ($P < 0.05$).

 © The Author(s)

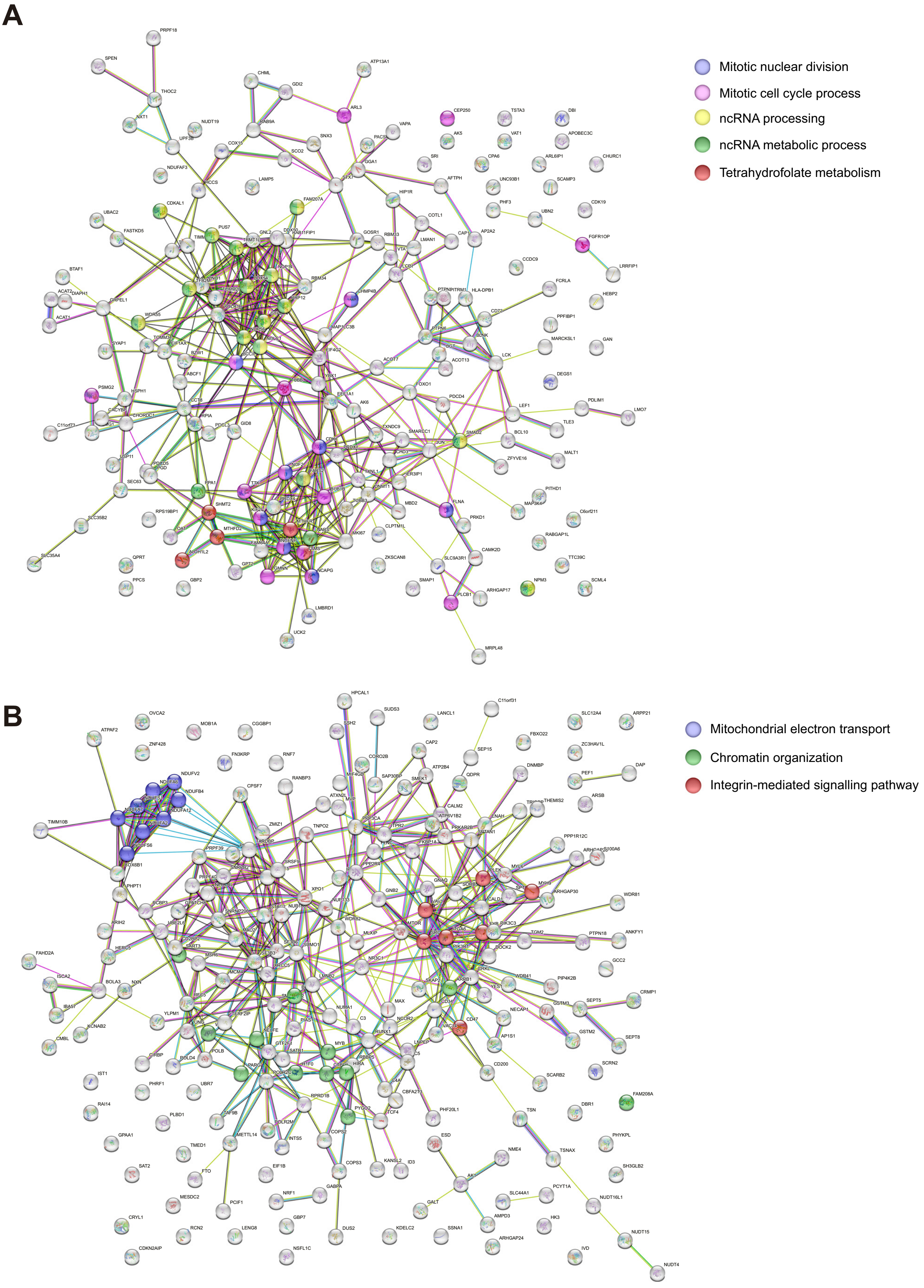
A
Mitotic nuclear division
Mitotic cell cycle process
ncRNA processing
ncRNA metabolic process
Tetrahydrofolate metabolism
B
Mitochondrial electron transport
Chromatin organization
Integrin-mediated signalling pathway

Figure EV4. Protein cluster analyses associated with CIN in cB-ALL.

(**A**, **B**) Protein–protein interaction network analysis with proteins positively (**A**) and negatively (**B**) correlated with CIN using the STRING database (version 11.5). Protein clusters are colored according with the indicated GO pathways. Protein–protein interaction (PPI) enrichment P-value $= 1 \times 10^{-16}$ (**A**) and 1.67×10^{-15} (**B**).

© The Author(s)

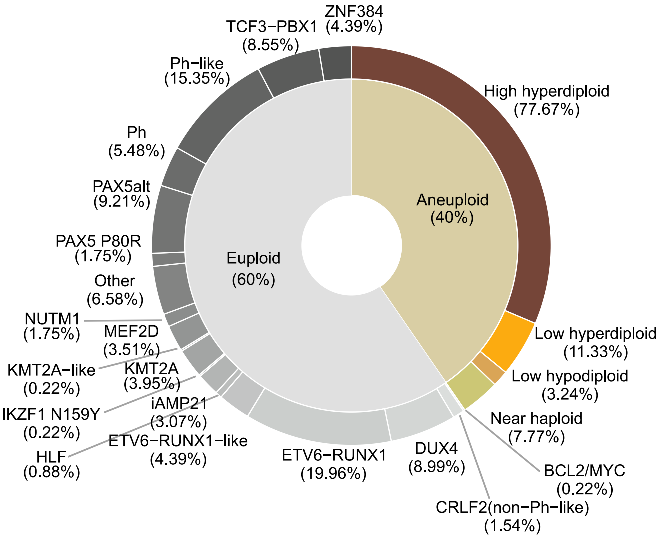

Figure EV5. Genetic subtypes of patients analyzed by RNA sequencing.

Pie chart depicting the frequency of individual cB-ALL molecular subgroups identified in the RNA-Seq St Judes hospital cohort of cB-ALL samples.

© The Author(s)
